# Prospective study of a group of pre-university students evaluating anxiety and depression relationships with temporomandibular disorders

**DOI:** 10.4317/jced.50745

**Published:** 2012-04-01

**Authors:** Ieda M. de Lucena, Luciane L F R. Rodrigues, Marcelo L. Teixeira, Daniel H. Pozza, Antonio S. Guimaraes

**Affiliations:** 1 Master’s Degree in Temporomandibular disorders, Dental Research Institute Sao Leopoldo Mandic; 2 PhD in Dentistry, Area of Concentration – Oral Physiology, UNICAMP University Professor, Dental Research Institute Sao Leopoldo Mandic; 3 PhD in Prosthodontics, University of São Paulo Professor, Dental Research Institute Sao Leopoldo Mandic; 4 PhD in Dentistry, Federal University of Bahia Professor, Department of Experimental Biology at the Medical School and IBMC, University of Porto; 5 PhD in Health Science, Professor, Dental Research Institute Sao Leopoldo Mandic

## Abstract

Objectives: The aim of this prospective longitudinal study was to evaluate the relationships between anxiety, depression, and temporomandibular disorders (TMD) in a sample of pre-university students submitted to a stressful event. Study Design: 153 students from a pre-university course (82 females and 71 males between 16 and 31 years old) were given a survey about TMD symptoms and a survey about anxiety and depression scale at the beginning and the end of the preparatory course (August 2009-T1, and November 2009-T2). Results: Results were analyzed using a chi-square test and Odds Ratio (OR), significance level of α = 0.05. Statistical significance were found to depression rates in students with TMD (16% on T1 and 26% on T2, p = 0.001) as well as in general sample (12% on T1 and 22% on T2, p = 0.009), anxiety and TMD symptoms presented constant rates in both periods. Increased risk of having TMD were found in participants with anxiety (OR 2.6 in T2 and 5.6 in T1) and depression (2.0 in T2 and 3.3 in T1), but only anxiety reach statistical significance in both periods. Conclusions: TMD symptoms were a fluctuating variable that exchange between some individuals of this study. Independently of the TMD, depression rates significant increased in the evaluated period. Finally, anxiety was the psychological symptom related to the increased risk of having TMD.

** Key words:**Temporomandibular disorders, anxiety, depression, orofacial pain, hospital anxiety and depression scale.

## Introduction

Temporomandibular disorders (TMD) was defined as mixes conditions involving two anatomical areas that covers a series of clinical problems that involve the masticatory muscles, the temporomandibular joint and associated structures (1). TMD appears more frequently in individuals with pronounced or disproportionate anxiety, depression, self-harm and/or somatization, and psychological factors may be both predispose and perpetrate TMD and orofacial pain (2-7).

Some individuals express high levels of anxiety by sustained muscular tension and perpetuating myofascial trigger points and together with parafunctional habits could give rise to TMD symptoms. Depression and chronic pain are closely related, with each able to be the cause and consequence of the other (8,9). The biopsychosocial model has been defended as an etiological theory of temporomandibular joint disorders, forcing the professional who sees the patient to consider not only the biological aspect, but also the psychosocial aspect (6,10,11).

The pain impulses can be enhanced or inhibited, modifying the patient’s pain, depending on the modulation performed by the central nervous system in the trigeminal spinal tract nucleus, reticular formation, descending inhibitory system and also by the action of the modulating psychological factors. Thus, the psychological conditions may increase or decrease the stimulus potential that the pain produced, although only the psychological modulation is not sufficient to eliminate the painful perception (10).

Given the multifactorial aetiology complexity of TMD especially related to psychosocial factors, this study aimed to determine where there is any association between the rate of anxiety and depression with TMD symptoms in a group of pre-university students submitted to a stressful event.

## Material and Methods

This prospective longitudinal study presented a sample consisting of a cohort of 153 students who are participants in a 4 month competitive pre-university course in a town in southern Brazil. The Dental Research Institute Sao Leopoldo Mandic approved this study and participants filled an individual informed consent.

The students responded to the temporomandibular disorders questionnaire recommended by the European Academy of Craniomandibular Disorders (12) and the questionnaire recommended by the Hospital Anxiety and Depression (HAD) scale (13) on two occasions: at the beginning (August 2009 = T1) and at the end of the preparatory course for college entrance exams (November 2009 = T2). The interval between the two data collections was 110 days. Our previous hypothesis was that students with high anxiety will be more likely to report TMD symptoms at time two.

The temporomandibular joint disorders symptoms questionnaire recommended by the European Academy of Craniomandibular Disorders includes four questions. If the student answers yes to one of these four questions, he/she is considered positive for TMD symptoms. The HAD scale contains 14 multiple-choice questions. They consist of two subscales, one for anxiety and the other for depression, with seven items each. The overall score in each subscale goes from 0 to 21. The cut off for the subscales is eight for anxiety and nine for depression. These questionnaires were chosen by validation, feasibility, easy interpretation and previous experience of the team.

Aiming to correlate temporomandibular joint disorders, anxiety and depression, the results were statistically analyzed using SPSS 18.0 to perform the Cochran chi-square test and Odds Ratio. A level of significance (probability of type I error) of α = 0.05 was used.

## Results

**Figure 1:**
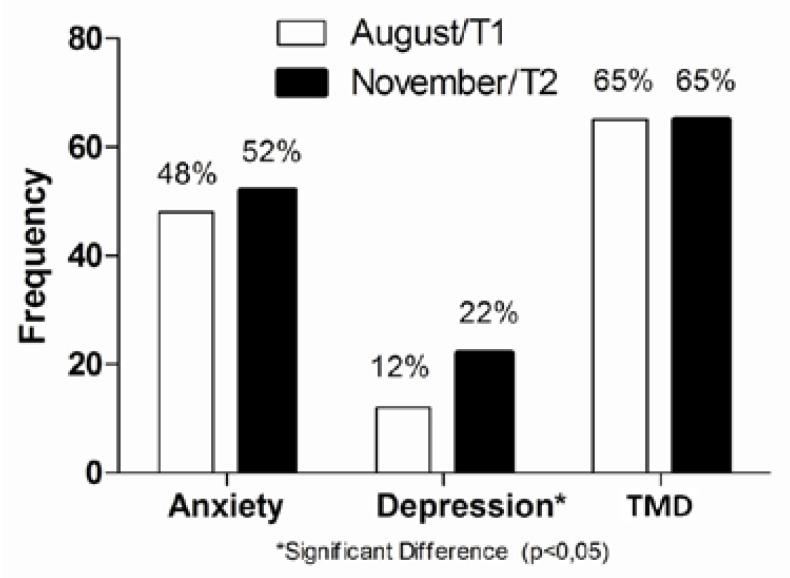
Rate of anxiety, depression and TMD symptoms.

Regarding gender, the sample of 153 students surveyed was composed of 82 female students (54%) and 71 males (46%). The age of the sample varied from 16 to 31 years old, with the mean being 18.2 ± 2.6 years. The anxiety rates for this sample were 48% (73 students) in T1 and 52% (80 students) in T2. The depression rates were 12% (19 students) in T1 and 22% (34 students) in T2. The TMD symptom rates were 65% (99 students) in T1 and in T2 (Fig. 1). In the Cochrane nonparametric statistical test, there was no significant difference between the two periods evaluated (T1 and T2) for the parameters of anxiety and TMD symptoms, however, there was a significant difference for depression in T1 and T2 (p = 0.009).After separating groups by TMD symptoms, it was verified that students with TMD presented more anxiety and depression than students without TMD, but these differences reach statistical significance only for anxiety ( Table 1). Participants with depression had increased risk of having TMD in T1 (OR = 3.3, 95% CI: 0.9, 11.8) and in T2 (OR = 2.0, 95% CI: 0.9, 4.9), that was more evident for that participants who had anxiety in T1 (OR = 5.6, 95% CI: 2.6, 12.0) and in T2 (OR = 2.6, 95% CI: 1.3, 5.2).

**Table 1:**
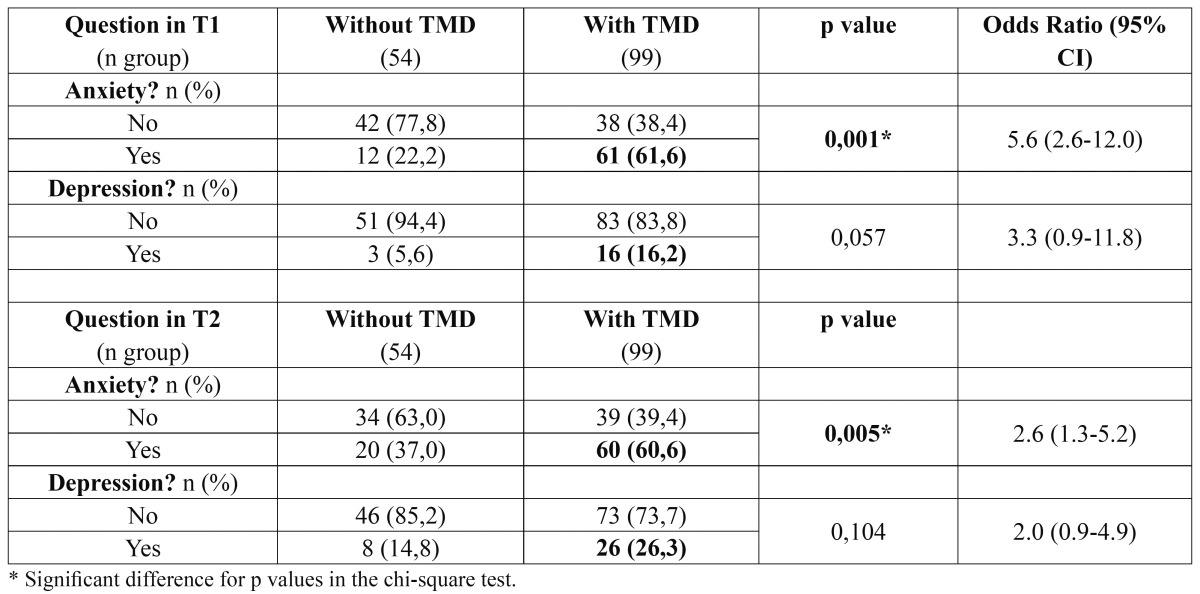
Longitudinal evaluation of fluctuation of the anxiety and depression in patients with or without TMD symptom in T1 (August, 2009) and T2 (November, 2009).

**Figure 2:**
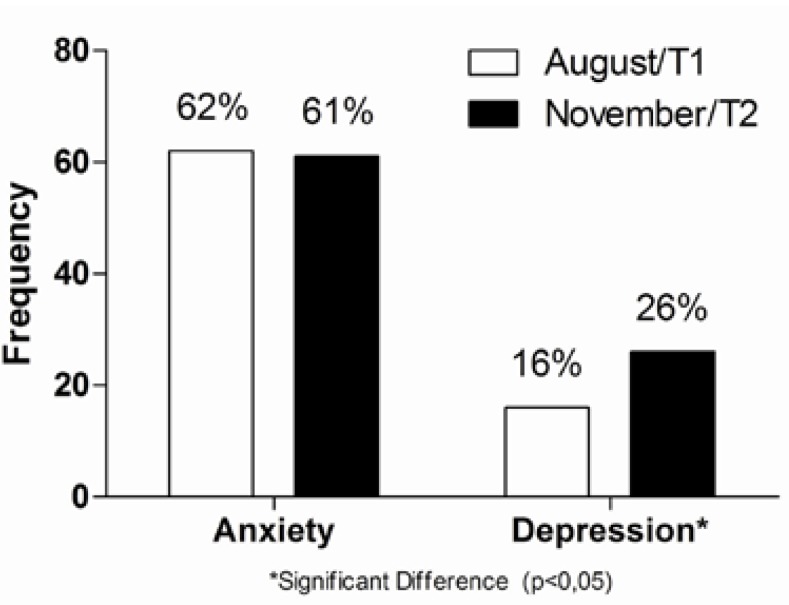
Rate of anxiety and depression in the group with TMD symptoms.

The anxiety rate of 99 students with TMD symptoms was 62% (61 students) in T1 and 61% (60 students) in T2 while the depression rate was 16% (16 students) in T1 and 26% (26 students) in T2 (Fig. 2). According to the Cochran nonparametric statistical test, there was no significant difference between anxiety in T1 and T2 for the patients with TMD symptoms. However, there was a significant difference for depression in T1 and T2 times (p = 0.001).

The longitudinal evaluation of TMD symptoms showed that 99 students had TMD symptoms at the beginning, in the first study (August 2009 – T1) and 99 students also had TMD symptoms at the end, in the second study (November 2009 – T2). However, as shown in  Table 2, this number does not correspond to the same individuals, since 17 of the 99 students that reported having TMD symptoms at the beginning of the study had remission of symptoms during the period evaluated and, coincidentally, the same number of students started having TMD symptoms in this period, giving the false impression that the same individuals has TMD symptoms during the whole evaluated period.

**Table 2:**
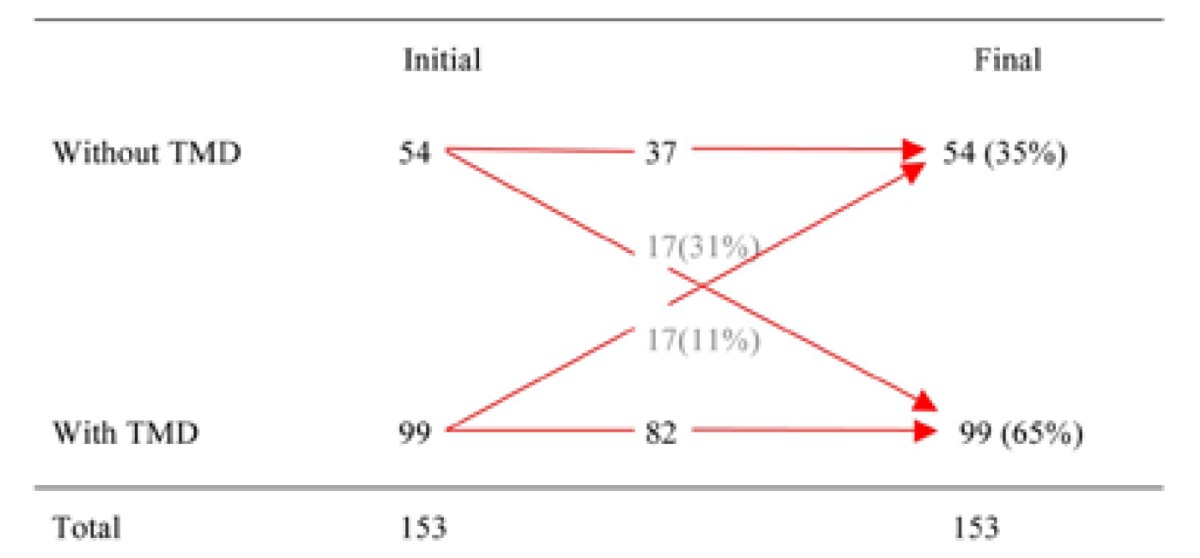
Longitudinal evaluation of fluctuation of the TMD symptom.

## Discussion

In this study, the frequency of 65% of TMD symptoms was similar to that found in other studies reported in literature, such as 63% (14) and 68% (15). However, other authors reported lower frequencies, varying from 3% to 50% (5,16-20). These discrepancies could be explained by the different methodology of data collection related to TMD signs and symptoms (16), or by separating TMD of articular origin from muscular pain (19), or even by differences in the sample, when cultural, economic and educational aspects are considered (20). We must also consider the fact that the present study sample was subjected to a sharp level of stress related to the national college entrance exam, which could have generated higher rate of TMD symptoms (7). Indeed the sample chosen was due to the stress that the students were under.

Another factor to be considered is that different populations have different prevalence of the same disease, like what happens in myofascial pain patients from tertiary care clinics, where numbers vary from 31% for Asians (18), to 38% (21) and 50% (22) for Italians, 69% and 82% for Israelis (23) and close to 76% for Americans and Swedes (24). The population of this study was formed of young people with an average age of 18 years old, and in the aforementioned studies the average age variation was between 34 and 41 years old, which may also contribute to these differences.

 In the present research, the anxiety rate of the group of students with TMD symptoms was very similar in the two periods, 62% in August of 2009 (T1) and 61% in November of 2009 (T2). The rate of depression in the group of students with TMD symptoms was 16% in T1 and rose to 26% in T2. These results are similar to those described by other studies in the literature, where the authors reported 58% for anxiety and 23% for depression (25), 15% moderate depression and 13% severe depression (19), 21% depression in the group of patients with joint pain, 26% in the group with myofascial pain and 25% in the group with myofascial and joint pain (26) and 19% of patients with severe depression (4). There are some differences in the results of this study when compared to the 40% for moderate and severe depression (18) or even the 25% for anxiety (27). These differences should not be credited only to methodological differences, but they may also be explained by differences in cultural, social and economic factors, education, the health of the researched population, and the fact that the questionnaires used to determine the rate of anxiety and depression in the studies mentioned are based on values of societies that are different from the individuals in this study (18,27).

It was verify that when compared to anxiety there was a weak correlation between depression and TMD symptoms, which is consistent with another study of the Brazilian population (2). However, several studies in the literature show a positive relationship between depression and TMD (4,5,7,9,10,18-20,25,26,28). Analyzing these articles, we can observe that all were related to groups of individuals with TMD signs and symptoms and some studies linked depression to TMD or chronic pain (7,9,25,28). These variables in the sample (TMD signs and symptoms and chronic TMD) may be an explanation for the difference in the results of the positive rela-tionship between depression and TMD in these studies. Another explanation may be related to the fact that the sample of this study was of students that were preparing to go to university and probably unlikely to present depression, since depression is a disease that reduces interest and pleasure to do these activities. Thus, it is assumed that the students with depression did not even register for the college entry preparatory course.

Another possible explanation lies in the average age of the individuals, with depression seeming to be a greater characteristic of the higher age brackets, while anxiety seems to be more related to acute stress (29). In this study we verified that there was a high association between anxiety and TMD symptoms. Many articles, even using different methodologies linking anxiety and TMD, showed a positive relationship between the two, which is consistent with this research (25,28).

Evaluating the longitudinal behaviour of the TMD symptoms in this study, we observed that there were the same number of individuals with TMD symptoms in T1 and T2, giving the impression that the group with TMD symptoms stayed the same during the evaluated period. However, analyzing it more closely (Table 2), we can see that there were a percentage of students that went from the group of TMD symptoms (31%) and a percentage of students that stopped having TMD symptoms (11%) during the period. This swing gives a fluctuating characteristic to TMD symptoms. This result is similar to other reported research (20,30), in addition to being longitudinal, the chart shows the movement of the individuals during the evaluated period and thus, the characteristic could be observed. It was also interesting to note in this study that the fluctuating characteristic of TMD symptoms happened in a short period of time. This finding helps to better understand the fluctuating behaviour of TMD symptoms, as well as plan a respective treatment. Thus, it allows us to speculate that some patients may show improvement in the TMD even without being treated by a health professional.

Although TMD symptoms presented the same rates in both periods, it was a fluctuating variable because while some individuals get free of symptoms, others become feeling TMD. Independently of having or not TMD, depression rates significant increased in the evaluated period, which was not observed for anxiety. Meanwhile, when analyzing both psychological symptoms, anxiety was more related to the increased risk of having TMD than depression.

## References

[1] Laskin DM (2008). Temporomandibular disorders: a term past its time?. J Am Dent Assoc.

[2] Bonjardim LR, Gaviao MB, Pereira LJ, Castelo PM (2005). Anxiety and depression in adolescents and their relationship with signs and symptoms of temporomandibular disorders. Int J Prosthodont.

[3] Ohrbach R, Dworkin SF (1998). Five-year outcomes in TMD: relationship of changes in pain to changes in physical and psychological variables. Pain.

[4] Celic R, Panduric J, Dulcic N (2006). Psychologic status in patients with temporomandibular disorders. Int J Prosthodont.

[5] Bonjardim LR, Lopes-Filho RJ, Amado G, Albuquerque RL, Goncalves SR (2009). Association between symptoms of temporomandibular disorders and gender, morphological occlusion, and psychological factors in a group of university students. Indian J Dent Res.

[6] Burris JL, Evans DR, Carlson CR (2010). Psychological correlates of medical comorbidities in patients with temporomandibular disorders. J Am Dent Assoc.

[7] Phillips JM, Gatchel RJ, Wesley AL, Ellis E (2001). Clinical implications of sex in acute temporomandibular disorders. J Am Dent Assoc.

[8] GaldOn MJ, Dura E, Andreu Y, Ferrando M, Poveda R, Bagan JV (2006). Multidimensional approach to the differences between muscular and articular temporomandibular patients: coping, distress, and pain characteristics. Oral Surg Oral Med Oral Pathol Oral Radiol Endod.

[9] Dworkin RH, Gitlin MJ (1991). Clinical aspects of depression in chronic pain patients. Clin J Pain.

[10] Davis CE, Carlson CR, Studts JL, Curran SL, Hoyle RH, Sherman JJ (2010). Use of a structural equation model for prediction of pain symptoms in patients with orofacial pain and temporomandibular disorders. J Orofac Pain.

[11] Lee LT, Yeung RW, Wong MC, McMillan AS (2008). Diagnostic sub-types, psychological distress and psychosocial dysfunction in southern Chinese people with temporomandibular disorders. J Oral Rehabil.

[12] De Boever JA, Nilner M, Orthlieb JD, Steenks MH (2008). Recommendations by the EACD for examination, diagnosis, and management of patients with temporomandibular disorders and orofacial pain by the general dental practitioner. J Orofac Pain.

[13] Botega NJ, Bio MR, Zomignani MA, Garcia C, Pereira WA (1995). Mood disorders among inpatients in ambulatory and validation of the anxiety and depression scale HAD. Rev Saude Publica.

[14] Otuyemi OD, Owotade FJ, Ugboko VI, Ndukwe KC, Olusile OA (2000). Prevalence of signs and symptoms of temporomandibular disorders in young Nigerian adults. J Orthod.

[15] Sonmez H, Sari S, Oksak Oray G, Camdeviren H (2001). Prevalence of temporomandibular dysfunction in Turkish children with mixed and permanent dentition. J Oral Rehabil.

[16] Conti PC, Ferreira PM, Pegoraro LF, Conti JV, Salvador MC (1996). A cross-sectional study of prevalence and etiology of signs and symptoms of temporomandibular disorders in high school and university students. J Orofac Pain.

[17] Macfarlane TV, Kenealy P, Kingdon HA, Mohlin BO, Pilley JR, Richmond S (2009). Twenty-year cohort study of health gain from orthodontic treatment: temporomandibular disorders. Am J Orthod Dentofacial Orthop.

[18] Yap AU, Dworkin SF, Chua EK, List T, Tan KB, Tan HH (2003). Prevalence of temporomandibular disorder subtypes, psychologic distress, and psychosocial dysfunction in Asian patients. J Orofac Pain.

[19] Rantala MA, Ahlberg J, Suvinen TI, Nissinen M, Lindholm H, Savolainen A (2003). Temporomandibular joint related painless symptoms, orofacial pain, neck pain, headache, and psychosocial factors among non-patients. Acta Odontol Scand.

[20] Egermark I, Carlsson GE, Magnusson T (2001). A 20-year longitudinal study of subjective symptoms of temporomandibular disorders from childhood to adulthood. Acta Odontol Scand.

[21] Manfredini D, Chiappe G, Bosco M (2006). Research diagnostic criteria for temporomandibular disorders (RDC/TMD) axis I diagnoses in an Italian patient population. J Oral Rehabil.

[22] Manfredini D, Piccotti F, Ferronato G, Guarda-Nardini L (2010). Age peaks of different RDC/TMD diagnoses in a patient population. J Dent.

[23] Reiter S, Eli I, Gavish A, Winocur E (2006). Ethnic differences in temporomandibular disorders between Jewish and Arab populations in Israel according to RDC/TMD evaluation. J Orofac Pain.

[24] List T, Dworkin SF (1996). Comparing TMD diagnoses and clinical findings at Swedish and US TMD centers using research diagnostic criteria for temporomandibular disorders. J Orofac Pain.

[25] Madland G, Feinmann C, Newman S (2000). Factors associated with anxiety and depression in facial arthromyalgia. Pain.

[26] Manfredini D, Bandettini Di Poggio A, Romagnoli M, Dell'Osso L, Bosco M (2003). A spectrum approach for the assessment of manic-depressive symptoms accompanying temporomandibular disorders. Minerva Stomatol.

[27] Saheeb BD, Otakpor AN (2005). Co-morbid psychiatric disorders in Nigerian patients suffering temporomandibular joint pain and dysfunction. Niger J Clin Pract.

[28] Glaros AG (2000-2001). Emotional factors in temporomandibular joint disorders. J Indiana
Dent Assoc.

[29] Marmorstein NR (2007). Relationships between anxiety and externalizing disorders in youth: the influences of age and gender. J Anxiety Disord.

[30] Magnusson T, Egermark I, Carlsson GE (2002). Treatment received, treatment demand, and treatment need for temporomandibular disorders in 35-year-old subjects. Cranio.

